# Editorial

**Published:** 1896-12

**Authors:** 


### Editorial.

### THE STATE LUNATIC ASYLUM.

The report of the Georgia Lunatic Asylum for the year ending September 1, 1896, is just from the press. During the past year there were 541 admissions, of which number 332 were whites, 209 colored. There were discharged, removed, died and eloped, 362 patients. There were in the asylum at the close of the year ending September 1, 1895, 1832 inmates, which added to the present number makes 2002, of which there were 1373 white and 629 colored. The per capita expenses were 35 cents per day. The Trustees recommend an increase in the amount of the appropriation to meet the steady increase in the number of the inmates. It is estimated that the number will approach 2200 in 1897, for the maintenance of whom $280,000 will be required. The facilities have been increased for extinguishing fires in the building, as owing to the character of the inmates great precautions in this direction are necessary. The Trustees further ask that the law on the question of admissions be so amended and altered as to allow insane citizens of other States to be temporarily received into the asylum in emergencies. The health of the inmates was generally good; the ratio of deaths to the number treated was eight per cent. The cause of death, which is often vaguely and loosely stated, was generally old age, apoplexy, exhaustion, and chronic diseases of various organs.

The causes of insanity have been tabulated, but are too indefinite to be of much real statistical value. For example, six cases are ascribed to "womb trouble," and three to "uterine trouble," nine to "the female trouble," and four to "spinal trouble." The interest that attaches to the etiology of insanity, especially among negroes, to say nothing of the sociological importance in the latter instance, renders desirable a more careful and explicit study than is implied in the general term, "trouble."

The acquisition of a salaried pathologist is a decided gain, and it is to be hoped that the legislature will not be niggardly in respect to ample appropriation for full equipment of this important department.

